# The utility of polysomnography for the diagnosis of NREM parasomnias: an observational study over 4 years of clinical practice

**DOI:** 10.1007/s00415-014-7578-2

**Published:** 2014-11-20

**Authors:** Chiara Fois, Mary-Anne S. Wright, GianPietro Sechi, Matthew C. Walker, Sofia H. Eriksson

**Affiliations:** 1 Department of Clinical and Experimental Epilepsy, UCL Institute of Neurology, National Hospital for Neurology and Neurosurgery, 33 Queen Square, Box 29, London, WC1N 3BG UK; 2 Department of Clinical and Experimental Medicine, University of Sassari, Sassari, Italy

**Keywords:** Polysomnography, Sleep study, NREM parasomnias, Arousal disorders, Sleepwalking, Epilepsy

## Abstract

Polysomnography (PSG) is considered the gold standard for diagnosis of non-rapid eye movement (NREM) parasomnias, however its diagnostic yield has been rarely reported. We aimed to assess the diagnostic value of polysomnography in different categories of patients with suspected NREM parasomnia and define variables that can affect the outcome. 124 adults referred for polysomnography for suspected NREM parasomnia were retrospectively identified and divided into clinical categories based on their history. Each polysomnography was analysed for features of NREM parasomnia or different sleep disorders and for presence of potential precipitants. The impact on the outcome of number of recording nights and concomitant consumption of benzodiazepines and antidepressants was assessed. Overall, PSG confirmed NREM parasomnias in 60.5 % patients and showed a different sleep disorder in another 16 %. Precipitants were found in 21 % of the 124 patients. However, PSG showed limited value when the NREM parasomnia was clinically uncomplicated, since it rarely revealed a different diagnosis or unsuspected precipitants (5 % respectively), but became essential for people with unusual features in the history where different or overlapping diagnoses (18 %) or unsuspected precipitants (24 %) were commonly identified. Taking benzodiazepines or antidepressants during the PSG reduced the diagnostic yield. PSG has a high diagnostic yield in patients with suspected NREM parasomnia, and can reveal a different diagnosis or precipitants in over 40 % of people with complicated or atypical presentation or those with a history of epilepsy. We suggest that PSG should be performed for one night in the first instance, with leg electrodes and respiratory measurements and after benzodiazepine and antidepressant withdrawal.

## Introduction

Parasomnias are undesirable physical events or experiences that occur during sleep [1] and are classified by the sleep stage from which they arise [1, 2]. NREM parasomnias arise from non-rapid eye movement (NREM) sleep, typically from slow-wave sleep (SWS), and include three typical behaviours–confusional arousals, sleep walking and sleep terrors. They are also termed “arousal disorders” since the episodes usually occur during the transition from SWS to an arousal phase or awakening [1–3].

NREM parasomnia typically occurs in childhood although an onset or persistence into adult life is not uncommon [1, 2, 4–7]. Their diagnosis is essentially clinical and often based on patient and bed-partner interviews. ICSD-2, and recently ICSD-3, propose essential diagnostic criteria for each of the three subtypes above [1, 2].

Typical NREM parasomnia is usually considered a benign condition, easy to recognise and treat. However, NREM parasomnia diagnosis can be challenging when the clinical history is unusual because of the age of onset, the time or duration of the episodes at night, the presence of suspected precipitating factors (such as periodic limb movements-PLMS or obstructive respiratory events), the unresponsiveness to conventional therapy or the occurrence of complex or dangerous behaviours [1, 8–12]. Indeed, on occasion, NREM parasomnia can be dangerous, resulting in injurious accidents or, not infrequently, can lead to sleep disruption [1, 10].

Clinical diagnosis may be especially challenging when there are similarities to other paroxysmal nocturnal events such as REM parasomnia or epilepsy [13–15], in particular in those patients in whom multiple conditions coexist [16–18].

During the last two decades increasing attention has been placed on clinical methods to distinguish nocturnal frontal lobe epilepsy from parasomnia [19–21], but, so far, no clinical algorithm or clinical questionnaire has been shown to differentiate reliably between these two different groups of sleep disorders [22–24]. Therefore overnight video-polysomnography (PSG) is still considered the gold standard test [23].

According to the American Academy of Sleep Medicine (AASM) [1], PSG can provide support for the clinical diagnosis of NREM parasomnia by documenting multiple arousals from SWS unaccompanied by parasomnia behaviours or strong support by documenting arousals from SWS accompanied by behaviours typical of arousal disorders.

Although PSG is often used to assist the clinician in the diagnosis of NREM parasomnia, the diagnostic value of the test has not been clearly established; there have only been a few studies [25–28] and some did not differentiate between parasomnia type [28].

The AASM published practice parameters for the indication of PSG for different sleep disorders, including parasomnia [11, 12].

However, the evidence supporting the guideline’s recommendations is somewhat conflicting and mainly based on small cases series and not always directly related to routine practice [11, 12, 29].

Moreover, the guidelines do not include information on how many nights of PSG should be performed and whether, or which kind, of drug discontinuations should be instigated before the overnight recording.

We aimed to define the diagnostic value of PSG among different clinical categories of patients referred for PSG to a single Sleep Centre for suspected NREM parasomnia, in order to assess how often PSG is able to facilitate the diagnosis, either confirming NREM parasomnia or ruling out alternative diagnoses or precipitant factors. Such information will help determine which groups of patients may benefit from an overnight recording and avoid unnecessary investigations of people in whom PSG will be of limited value.

We also aimed to clarify the confounding effect of concomitant consumption of the most widely used drugs for NREM parasomnia (antidepressants and benzodiazepine receptor agonists) on PSG results and to establish the utility of performing more than one night of recording in order to increase the diagnostic yield.

## Methods

### Patients and clinical categories

Medical records of a total of 592 consecutive patients who underwent one or more sleep studies at the National Hospital for Neurology and Neurosurgery over a period of 4 years (January 2009–December 2012) were retrospectively reviewed.

Among them, 126 patients were referred for PSG for suspected NREM parasomnia by sleep specialists, whereas 466 were patients referred for PSG for other types of sleep disorders.

Of 126 patients referred for suspected NREM parasomnia only patients who underwent one or two nights of diagnostic PSG were considered. Patients who underwent more than two nights of recording or repeated the study after having already been diagnosed with NREM parasomnia were excluded from the analysis. For patients that underwent more than one PSG study within the period above, only the first study was considered.

Therefore two patients were excluded from the recruitment, one because of three nights recording and another because this was a follow-up PSG in a patient already diagnosed with NREM parasomnia.

124 patients and consequently as many sleep studies were analysed. Based on the clinical history at the time of presentation and according to the ICSD-2 clinical criteria [1] the 124 patients were divided into four main categories (Table 1).

**Table 1:** Clinical categories made to group 124 patients referred for suspected NREM parasomnia, according to ICSD-2 clinical criteria

Clinical categories
(1) **Typical, uncomplicated NREM parasomnia**
For time and duration of the episodes at night
Age at onset ≤16 years
(2) **Typical NREM parasomnia but complicated by**
(a) Injurious behaviour
(b) Precipitants (OSAS, PLMS)
(c) Unresponsiveness to conventional therapy
(3) **Unusual/atypical NREM parasomnia, not clinically defined because of**
(a) Age at onset >16 years
(b) Thought to be seizure related
(c) Overlapping features with other sleep disorders (RBD/narcolepsy/psychiatric/insomnia).
(4) **History of epilepsy in addition to suspected NREM parasomnia**

*OSAS* obstructive sleep apnoea syndrome, *PLMS* periodic limb movements syndrome, *RBD* REM sleep behaviour disorder

Frequency of the presumed NREM parasomnia episodes was not considered as a factor against the clinical diagnosis of NREM parasomnia when the history was otherwise typical.

An arbitrary cut off of 16 years of age was chosen to differentiate childhood and youth onset of the episodes from an onset in adult life.

### Sleep analysis

All the sleep studies were carried out and scored by experienced clinical physiologists (neurophysiology) and then by clinicians specialising in sleep disorders, according to the AASM “Manual for the Scoring of Sleep and Associated Events” [30].

All patients underwent polysomnography using full 10–20 electrode placement [31] with additional polygraphy electrodes including bilateral electrooculogram referred to cross-linked mastoid electrodes, bilateral masseter EMG, submentalis EMG and bilateral tibialis anterior EMG. Pulse oximetry and airflow were also recorded if clinically indicated. The data were acquired and analysed using Nicolet (Viasys) equipment and software. The data were scored in 30-s epochs, and classified as light sleep, deep sleep, REM sleep or movement. Oxygen desaturations were noted as significant if there was a drop of more than 4 % from baseline. Episodes of hypopnoea or apnoea were analysed.

All arousals or awakenings were annotated. We scored as arousal each event characterised by an abrupt shifting of EEG frequency into alpha, theta or faster frequency, other than spindles, that lasted at least 3 s, according to the AASM “Manual for the Scoring of Sleep and Associated Events” [30]. Arousals were classified as spontaneous when not determined by any clear underlying event such as noise, light or other environmental disturbances.

Each sleep study was classified as decisive (arousals from SWS accompanied by NREM parasomnia behaviours), supportive (spontaneous arousals from SWS accompanied by more subtle behaviours such as raising the head, sympathetic activation, such as tachycardia, rhythmic delta activity on EEG but no epileptiform activity) or inconclusive (none of the above) for diagnosis of NREM parasomnia [1].

Alternative and overlapping diagnoses were also investigated based on the events recorded during the overnight study according to ICSD-2 [1].

Diagnosis of epilepsy was made if unequivocal interictal epileptiform EEG abnormality or clear epileptic events were captured.

We also identified any precipitant event such as PLMS or respiratory events as clinically significant when they demonstrated to have a clinical impact on the patient’s sleep stability, either causing an arousal or awakening or sleep stage shifting.

### Assessment of other variables

Concomitant consumption of benzodiazepine-receptor agonists (BZD or newer BzRAs) or any type of antidepressant (serotonin reuptake inhibitors-SSRIs, tricyclic antidepressants and serotonin–norepinephrine reuptake inhibitors-SNRIs) at the time of the sleep study was also investigated for each patient and correlated with outcome of the sleep study.

The utility of performing a second night recording, to increase the yield of the study, was also evaluated in patients that underwent two nights of recording. In particular, we looked at the episodes recorded over the first and the second night and classified as diagnostic the night on which we captured any clear, typical and useful event for the diagnosis. If one or more clear and typical episodes were present over both nights, we classified as diagnostic only the first night when the second night recording did not add any further information compared to the previous night.

### Statistical analysis

Statistical analysis was performed using the SPSS V.22 statistical software package.

Fisher’s exact test was used to test correlations between outcome (positive–negative) and other categorical variables such as main categories belonging (1, 2, 3, 4), age at onset (> or ≤ of 16 years), antidepressant/BDZ consumption (yes or no) and nights of recording (one or two).

Fisher’s exact test was also used to test correlation between presence of overlapping/different diagnoses or precipitants (yes or no) and main categories belonging (1, 2, 3, 4). Significance thresholds were set at *p* < 0.05. All tests were two tailed.

## Results

### Population

Among 124 patients referred for an overnight sleep study with a query of NREM parasomnia, there were 60 males and 64 females, with a mean age at time of consultation of 37 ± 1 years (range 18–80 years) and a median age of 34 years.

Most of the patients belonged to the third category (*n* = 73). This was either due to unusual features such as the age at onset >16 years (category 3.a, *n* = 12); the episodes were thought to be seizure related (category 3.b, *n* = 16); or the diagnosis was not clearly discernable due to a history suggestive of overlapping features with other sleep disorders such as REM parasomnia, narcolepsy, insomnia or psychiatric disturbance (category 3.c, *n* = 45).

A minority of patients belonged to the first and the second category (*n* = 39) for which the diagnosis was clearly delineated from the history alone; either patients with uncomplicated NREM parasomnia (category 1, *n* = 21) or patients with complicating associated features, such as injurious behaviour (category 2.a, *n* = 10) or suspected precipitants (category 2.b, *n* = 8).

None of the patients recruited fell within the category 2.c, since none of the patients were referred to us due to failure to respond to conventional therapy.

There were 12 patients already diagnosed with epilepsy but presenting with episodes at night suggestive of NREM parasomnia behaviours (category 4), (Tables 1, 2).

**Table 2:** Demographics and NREM parasomnia PSG outcome for each clinical category

Categories	Female (%)	Onset ≤16 years (%)^c^		PSG outcome				
				Mean age at admission^a^	Decisive (%)	Supportive (%)	Inconclusive (%)	Total
1	61.9	100.0		31.1 ± 1 (22–49)	42.8	52.4	4.8	21
2.a	30.0	100.0		27.9 ± 2 (20–43)	50.0	10.0	40.0	10
2.b	50.0	100.0		37.1 ± 4 (26–61)	37.5	25.0	37.5	8
2.c^b^	NA	NA		NA	NA	NA	NA	NA
3.a	66.7	0.0		41.4 ± 3 (28–67)	58.3	8.3	33.3	12
3.b	43.7	62.5		35.8 ± 3 (21–58)	43.8	31.2	25.0	16
3.c	46.7	44.1		41.7 ± 2 (18–80)	20.0	22.2	57.8	45
4	66.7	45.4		34.3 ± 2 (20–52)	16.7	25.0	58.3	12
Total	51.6	60.3		37.0 ± 1 (18–80)	33.9	26.6	39.5	124

^a^Data are given as mean ± SEM. Range is given within brackets

^b^None of the patients recruited fell within this category

^c^This information was missing for three patients

### NREM parasomnia findings

Overall PSG was decisive or supportive for a diagnosis of NREM parasomnia in 75 of 124 overnight sleep studies analysed (60.5 %) (Table 2).

As expected, PSG was positive for NREM parasomnia (decisive or supportive) in a high proportion of cases with typical clinical history, in particular when uncomplicated (category 1; 95.2 %) but also in cases with typical but complicated NREM parasomnia (category 2; 61.1 %).

Conversely within the categories with an atypical or unusual history (category 3) or with a suspected coexistence with epilepsy (category 4), NREM parasomnia was found in a relatively lower proportion of patients (53.4 % and 41.7 %, respectively).

**Fig. 1:**
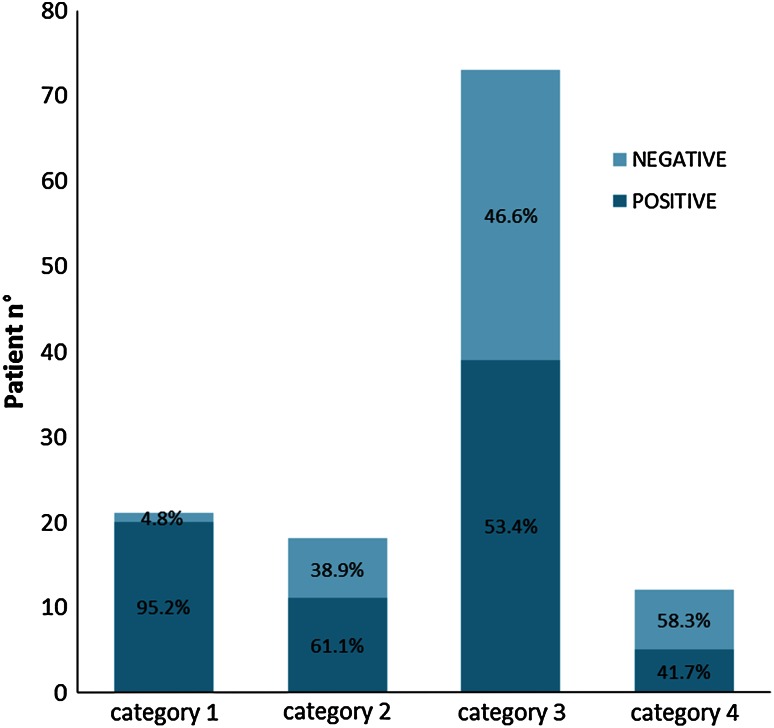
The PSG results significantly varied among the categories and the probability of having a test positive for NREM parasomnia decreased with the uncertainty of the clinical diagnosis (*p* = 0.001)

Therefore the probability of having a sleep study positive for NREM parasomnia differed among the categories, in keeping with the certainty of clinical diagnosis (*p* = 0.001, Fisher’s exact) (Fig. 1).

### Age at onset

Although NREM parasomnias are typically thought to start in childhood, the proportion of patients who had a PSG positive for NREM parasomnia was similar for patients with an onset before (67 %) or after (55 %) 16 years of age.

### Different/overlap diagnoses and precipitants findings

Among all 124 sleep studies analysed we found 9 (7.2 %) overlapping diagnoses, where PSG showed features of NREM parasomnia and another disorder (such as REM behaviour disorder-RBD or epilepsy) and 11 (8.9 %) differential diagnoses, where PSG was not supportive of NREM parasomnia but was instead suggestive of a different sleep disorder.

We found a high proportion of potential precipitants such as PLMs and obstructive respiratory events (hypopnoea or apnoea) during sleep, which were deemed to be clinically significant in 26 patients (21 %).

Overlapping, differential diagnoses or precipitating factors were rare (9 %) within the category of clinically typical NREM parasomnia (category 1), whereas they were frequent (43 %) in the other categories (categories 2, 3 and 4 together, *p* < 0.005) (Table 3).

**Table 3:** Different/overlap diagnoses and precipitants found on PSG for each clinical category

Categories	Total of patients per category	Patients with overlap diagnoses	Patient with different diagnoses	Patients with precipitants	Total of precipitants, overlap and different diagnoses (%)
1	21	1 (RBD)	0	1	9.5
2.a	10	0	1 (NAA)	1	20.0
2.b	8	0	0	2	25.0
2.c	0	0	0	0	0.0
3.a	12	0	1 (PD)^a^	3	33.3
3.b	16	3 (1 RBD;2 E)	1 (E)	2	37.5
3.c	45	2 (RBD)	7 (5 RBD;1 NAA; 1 E)	12	46.7
4	12	3 (E)	1 (PD)^a^	5	75.0
Total	124	9	11	26	37.1

*NAA* nightmare-associated arousal, *E* epilepsy, *RBD* REM sleep behaviour disorder, *PD* psychiatric disturbance

^a^Dissociative state and panic attack, respectively

### Benzodiazepine and antidepressant consumption

26 out of 114 patients were taking BDZ and/or antidepressants (*n* = 5 BDZ; *n* = 10 SSRI; *n* = 2 tricyclics; *n* = 6 SSRI and BDZ; *n* = 3 SSRI and tricyclics) at the time of the sleep study. In 17 (65.4 %) of the 26 patients taking the above medication, PSG did not show any features diagnostic of NREM parasomnia (Table 4).

**Table 4:** Polysomnography outcome related to drug consumption in 26 patients taking potential outcome-affecting drugs

*n* = 26 patients taking potential outcome-affecting drugs^a^	BDZ	SSRI	SNRI	Tricyclics	NREM parasomnia PSG outcome
1	X	X			NEG
2		X		X	NEG
3		X			NEG
4	X				POS
5	X	X			POS
6		X		X	NEG
7		X			POS
8	X				NEG
9	X	X			POS
10		X			POS
11				X	POS
12				X	POS
13		X			POS
14	X				NEG
15	X	X			NEG
16		X			POS
17		X		X	NEG
18	X	X			NEG
19		X			NEG
20		X			NEG
21	X				NEG
22	X	X			NEG
23		X			NEG
24	X				NEG
25		X			NEG
26		X			NEG

*BDZ* benzodiazepine-receptor agonists, *SSRI* serotonin reuptake inhibitors, *SNRI* serotonin–norepinephrine reuptake inhibitors, *PSG* polysomnography, *POS* positive, *NEG* negative

^a^This information was missing for 10 patient out of 124

We found that a concomitant consumption of antidepressants and/or BDZ at the time of the sleep study affected the outcome and significantly correlated with the probability to have a negative PSG (*p* = 0.01).

### Diagnostic night evaluation

28 out of 124 patients studied were scheduled for and underwent 2 nights of PSG. We found that 18 out of the 28 patients recorded for 2 night (64.2 %) had a positive PSG (*n* = 12 NREM parasomnia alone; *n* = 4 overlap diagnoses; *n* = 2 different diagnosis) compared to the 68 (70.8 %) positive PSG out of 96 patients recorded for one night only.

Therefore, we did not find any significant statistical correlation between PSG outcome and number of recording nights (*p* = 0.512).

Further, in 15 of the 18 patients above, the diagnosis was made on the first night since the second night was not diagnostic (*n* = 2) or did not to add any further information compared to the previous (*n* = 13). Only in three studies was the second night decisive for diagnosis (Table 5).

**Table 5:** Diagnostic night evaluation for 28 patients with two consecutive recording nights

*n* = 28 patients that underwent two recording nights	Category belonging	NREM parasomnia found	Other diagnoses found	Diagnostic night
1	1	x		I–II
2	2a	x		II
3	2a			
4	3a	x		I
5	3a	x		I–II
6	3b	x	x (E)	II
7	3b		x (E + exaggerated hypnic jerks)	I
8	3b	x	x (E)	I–II
9	3b	x		I–II
10	3b	x		I–II
11	3b	x		I–II
12	3c			
13	3c	x		I–II
14	3c	x		II
15	3c	x		I–II
16	3c			
17	3c		x (E)	I–II
18	3c			
19	3c	x		I–II
20	3c			
21	3c			
22	3c	x	x (RBD)	I–II
23	4			
24	4			
25	4	x		I–II
26	4			
27	4			
28	4	x	x (E)	I–II

*E* epilepsy, *RBD* REM sleep behaviour disorder, *I* first, *II* second

## Discussion

Polysomnography is a widely used diagnostic tool to assist in the diagnosis of sleep disorders.

There is limited evidence regarding the utility of performing PSG for the diagnosis of NREM parasomnia.

It has been two decades since Aldrich and Jahnke’s [28] paper on the diagnostic value of PSG in patients with suspected parasomnia.

They retrospectively studied 65 children and 57 adults presenting with parasomnia-like episodes (both NREM and REM) and found that PSG was overall useful and gave diagnostic information in 65.5 % of patients. They concluded that for patients with unexplained nocturnal movements or behaviour, either with or without known epilepsy, the diagnostic yield with PSG is substantial. In particular, Aldrich and Jahnke emphasised the superiority of video PSG, compared to standard PSG without video recording, to correlate behaviour with EEG while evaluating parasomnias patients. Apart from this, only small case series or case reports have been reported [25–27].

The lack of data regarding the role of PSG for this group of people has led to a great divergence in clinical practice, among different Sleep Centres, regarding the indications for performing overnight sleep studies.

With this study, we assessed the utility of PSG in different clinical categories of patients with suspected NREM parasomnia trying to clarify when this investigation should be used and which measures should be adopted.

We found that, as previously suggested by Aldrich and Jahnke [28], PSG combined with video-monitoring has a high diagnostic value in people with suspected NREM parasomnia giving the diagnosis in 76.5 % of the 124 studied patients. In particular, it confirmed the NREM parasomnia diagnosis in 60.5 % of the cases and a different or overlap diagnosis in 16 % of them, most of which were found to be RBD (*n* = 9) or epilepsy (*n* = 7).

However, the value of PSG varied among the clinical categories.

We found that when the diagnosis was clear and history uncomplicated, PSG confirmed NREM parasomnia in almost all patients (95 %) and very rarely showed any underlying unsuspected precipitants or different sleep disorders (5 %, respectively). It is of course also possible that the patients with PSG did not identify any NREM parasomnia might not have the disorder.

Conversely in people in whom the diagnosis was not clinically obvious or the history showed some unusual features, the probability of having NREM parasomnia confirmed by PSG was significantly lower (53 %) but the presence of unsuspected or unrecognised different diagnoses (18 %) or potential precipitants (24 %) was relatively high. Thus, whereas PSG has a limited value in typical NREM parasomnias, it is advisable for people in whom there is clinical uncertainty due to unusual features in the history.

We also found that adulthood age of onset of NREM parasomnia, often considered as an unusual feature [12], did not affect the probability to find NREM parasomnia in the PSG study. In fact NREM parasomnia was found in a similar proportion of people with childhood (67 %) or adulthood onset of the disorder (55 %). Therefore, age at onset should not to be considered alone as a determining feature against the clinical diagnosis.

Patients under investigation for suspected NREM parasomnia often undergo more than one night of recording in order to increase the probability of capturing events; this is particularly so in those patients who are more challenging or present with infrequent episodes. This is time and resource consuming and also leads to increased inconvenience for patients. The advantage of longer recordings has never been sufficiently investigated. In our group of patients, we found that the second night rarely provided any additional information since the proportion of positive tests in people that underwent one night of recording (70 %) was similar to that found in people with two nights of PSG (64 %). In patients where the diagnosis was made, this was usually already clear from the first night of the recording (83 %). A single night’s admission would hence appear to be sufficient for the majority of patients.

For some patients in our study, no episodes were recorded even during longer admissions. Home video-recording has recently been also shown to be an alternative, useful tool on some occasions, in particular for people with questionable or inconclusive PSG or where no episodes are recorded in hospital, helping in appreciating the severity of the episodes and for ruling out stereotyped behaviours [8, 32].

NREM parasomnia episodes can be precipitated by external and internal factors in predisposed individuals. In general, any factor that increases the proportion of SWS (sleep deprivation or substances use) or sleep fragmentation with frequent arousals can facilitate events.

Obstructive respiratory events and PLMS have been reported in NREM parasomnia patients [1, 6, 16, 25, 33, 34] as potential precipitating factors often causing arousal or sleep disruption.

We found that, such comorbidities are not an infrequent finding in adult patients evaluated for NREM parasomnia (21 %) even in apparently asymptomatic patients.

Hence, the concomitant occurrence of PLMS or obstructive respiratory events in this group of people should not be underestimated, but their presence should be sought by routine monitoring of leg EMG channels and respiratory parameters.

Pharmacological treatment for NREM parasomnia is usually not necessary for typical, infrequent and non-injurious episodes. On the other hand, treatment may be required for people in whom the episodes cause discomfort or distress, such as excessive day-time somnolence or injuries to themselves or bed-partner.

The most widely used drugs for treatment of NREM parasomnia, when needed, are benzodiazepine and non-benzodiazepine hypnotics (i.e. clonazepam, diazepam, triazolam, zolpidem, etc.), tricyclics antidepressants (i.e. imipramine) and SSRIs (i.e. paroxetine, trazodone).

Nevertheless the effect of these drugs on NREM and NREM parasomnia remains controversial and their use is mainly based on case reports since large randomised control trials have still not been made [35, 36].

We found that concomitant consumption of hypnotics, tryciclics or SSRIs at the time of the recording significant correlated with a negative PSG result (*p* = 0.01), reducing the possibility to capture the events and therefore the yield of the test.

There are a number of limitations to our study. First, it is a retrospective study and thus clinical categorisation was based on previously documented clinical histories that might depend on different clinician experience and perspectives.

For the same reason, information was missing for a few patients regarding age of onset (*n* = 3) or drug consumption (*n* = 10).

Patients were recruited from a tertiary specialty clinic and may not be representative of all patients with NREM parasomnias. Further, in the absence of validated methods for detecting NREM parasomnias with PSG, the prevalence of suggestive features in our population may be overestimated; for example, spontaneous arousals from deep sleep are characteristic but not specific of this disorder.

Although the role of PSG for diagnosis of NREM parasomnias is also relevant for children, care should be taken in translating our findings to the pediatric population. Indeed in children, the proportion of typical NREM parasomnias is higher and certain alternative diagnoses such as RBD are far less common. Therefore the proportion of different diagnoses found on PSG might be lower. However, in unusual cases, PSG is critical to rule out epilepsy and precipitants in selected patients.

In conclusion, polysomnography has a high diagnostic yield in adults with suspected NREM parasomnia and is particularly useful to assist the diagnosis in people with an unusual or complicated history. Hence, when the clinical presentation alone does not allow a clear diagnosis an overnight evaluation of the episodes with comprehensive video-monitoring is essential. This should be performed for one night only in the first instance, with leg electrodes and measurement of respiratory parameters, and after benzodiazepine and antidepressant withdrawal, if at all possible.
